# Wet labs: A useful tool in training surgical residents in a third world country

**DOI:** 10.1016/j.amsu.2020.07.014

**Published:** 2020-07-16

**Authors:** Hina Inam, Narmeen Asif, Abdul Ahad Sohail, Saulat Hasnain Fatimi

**Affiliations:** a Department of Cardio-thoracic Surgery, Aga Khan University Hospital, Pakistan; b Aga Khan University Hospital, Pakistan

## Abstract

• Wet labs are a useful, cost-effective and safe tool in teaching of Cardiothoracic Surgery residents.
• In a third world country where advance real life simulators are not available.
• It improves resident's tissue handling and surgical skills.
• Allows faculty members to give continuous feedback to their residents.

## 1 Introduction

WATCH ONE, DO ONE, TEACH ONE is a known dictum reflecting the old tradition of surgical training, where residents and trainees were expected to not only perform but also teach a surgical procedure after seeing it once [1]. However this may not be applicable in cardiac surgery. Cardiac surgery being time sensitive, having no margin of error, presence of extremely sick patients and performance of complicated and technical procedures including microsurgical techniques and macro procedures makes this impossible.

However a paradigm shift has been seen in the surgical training of residents and trainee.

William Halsted was a pioneer of surgical training and spearheaded the first ever residency program in John Hopkins USA, in 1890. At that time surgical training was composed of long hours of observing and retracting in the operation theatre. However just operating theater may not be an ideal location for early training of surgical residents due to growing ethical concerns, time constraints in the operating theater, changes in resident work hours, requiring more structured training, physician burnout and learning curve associated risks [[2], [3], [4]].

Many studies report effective learning when trainees are involved actively in the process. This can include making notes, diagrams, being asked questions and being involved in the procedure being performed. However one retains only 5% of what has been taught via lectures [5]. As per studies modern day advancements like wet lab, dry lab and simulators show enhanced performance and better results [6]. Extensive work had been done upon this in the field of Ophthalmology which requires precision and excellent hand eye coordination for its microsurgical techniques with Alwadani et al. describing goat's eye as an ideal tissue for ophthalmological wet labs and hence describing wet labs an effective method to empower trainee surgeons to safely perform complex procedure with lower complication rates and decreasing their learning curve [7,8].

Wet labs using animal organs offer essential training and practice outside the operating theater. It is an important way to assess residents on the basis of their anatomy, concepts, instrument and tissue handling and skills. Wet lab may come across as an ethical concern as far as using live animals are concerned, however it is something greatly valued by medical students and residents [9]. The anatomical variations in animal organs help nurture critical thinking and helps recall in a risk-free environment. The wet lab provides a different preview for each participant; students get the chance to learn basic surgical skills and techniques, whereas the trainees and the consultants can perform surgical procedures like coronary anastomosis, segmental resection of lung lobes etc. Bovine and swine tissues are the most common animal organs that are taken for this teaching. Some countries make use of sheep and dogs as teaching organs for wet lab purposes.

Simulation and animal laboratory experience have been used extensively in cardiothoracic surgery research and training. Fann and coworkers [10] discussed about the use of porcine hearts in resident training. They also evaluated cardiothoracic surgery residents’ performance after one week of practice on task stations and beating heart simulator showing about 20% decrease in time for residents to perform anastomosis on task stations and 15% decrease in time on beating heart simulators, hence proving that practice outside the operation theatre is essential, safe and effective way of improving surgical skills for cardiothoracic surgery residents [10]. Divisi et al. describes the use of swine model in wet labs for VATS (Video Assisted Thoracoscopic Surgery) lobectomy and simulation based learning to decrease the residents learning curve during their course of training [11].

### 1.1 Purpose of this document

This letter to editor details the technicalities of a wet lab, its advantages. What it takes and how it is done. The technical proof of concept is the first phase in development of wet lab as an integral part in the curriculum of residents learning.

### 1.2 Rationale behind the concept

Training of residents in patients is time consuming and may be harmful for patients and therefore Wet labs can help train these residents. They are able to develop skills which are helpful in operating rooms.

## 2 Material and methods

The Department of Cardiothoracic Surgery at the Aga Khan University Hospital initiated Wet labs in anatomy dissection halls and was able to conduct these bimonthly workshops in valve repair, replacements and coronary anastomoses.

**Fig. 1:**
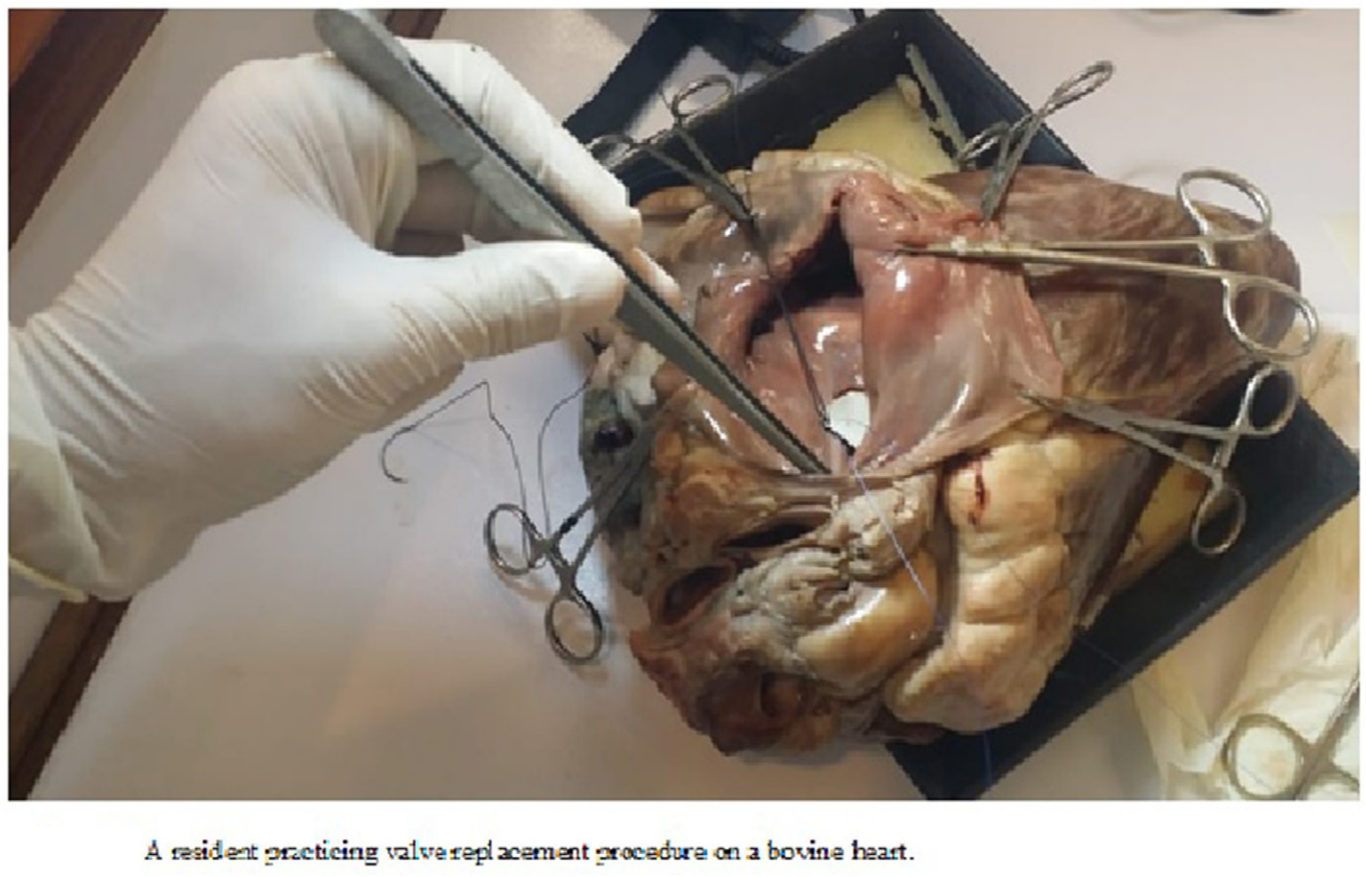
A resident practicing valve replacement procedure on a bovine heart.

It is easy to establish. We get our Bovine Hearts from a butcher at a minimal cost of a few hundred Pakistani rupees. We have dissecting wax trays in our dissection hall. We use old sutures or sutures that have been opened in the operation theatre but not used. We use veins saved in formalin from our coronary artery bypasses. Every resident has their own set of basic instruments (scissors, forceps, needle holder, hemostats) which they bring with them to each wet lab (Fig. 1).

Each wet lab session had been facilitated by a faculty member (who moderates the session and provides basic background knowledge on the topic that had been chosen for that particular session), while all other faculty members and consultants teach and supervise the residents on one to one basis at each table. The number of residents attending these sessions are all previously trained in general surgery and range from year 1 to year 4 cardiac surgery residents with approximately 5–7 residents in each session depending on their availability. The residents are evaluated on the basis of their anatomical knowledge, tissue handling and technical skills pre and post session.

### 2.1 Performance assessment

The residents are directly supervised by a faculty cardiac surgeon and chief resident during the entire session; continuous feedback is given to the resident regarding graft handling and orientation, instrument use and suture placement during the entire session. After completion, the anastomoses/valves are inspected by the moderator and additional feedback is given to the resident and the faculty member supervising that particular resident.

### 2.2 Observations

The residents were observed to become well versed and confident when it came to tissue handling, anastomosis and anatomical relationships of the heart even when working in the operation theatre.

### 2.3 Strengths

This is a single center experience with wet lab used as an integrated tool for residents teaching. Such wet labs are carried out regularly in the western countries and have shown remarkable results.

We have been able to conduct three successful workshops here at Aga Khan University Hospital on Valve Sparing Aortic root replacement, Segmental lobectomy of lung, mitral valve replacement, atrial and ventricular septal defect closures. All were attended by well renowned national and international cardiac surgeons. Also this is the first time such wet labs had been conducted in our institute and no other department in surgery had ever conducted it previously in our institute. To the best of our knowledge, no other studies or literature is available from our region that describes the use of wet labs or simulation based teaching of cardiac surgery residents in third world country.

## 3 Conclusion

Conducting wet labs is a cost-effective strategic teaching tool for residents which not only inculcates confidence in their surgical skills but also is helpful in teaching advanced skills at consultant's level and can greatly enhance surgical residents training and skills especially in third world countries where expensive simulators are not available in training institutes for residents practice.

## Ethical approval

Research studies involving patients require ethical approval. Please state whether approval or exemption has been given, name the relevant ethics committee and the state the reference number for their judgement. Please give a statement regarding ethnical approval that will be included in the publication of your article, if the study is exempt from ethnical approval in your institution please state this. I am submitting this Letter to Editor which is exempted from ethical approval in Aga Khan University Hospital.

## Source of funding

None.

## Declaration

Declaration: This manuscript has neither been presented nor published elsewhere before. However, the abstract has been published previously as part of conference abstract in CHEST regional congress (Athens 2019) by the same author under the title: ‘WET LAB AS A TOOL FOR TEACHING CARDIO THORACIC SURGERY RESIDENT: WHAT WOULD HALSTED SAY? [12].

## CRediT authorship contribution statement

**Hina Inam:** Conceptualization, Writing - original draft, Writing - review & editing. **Narmeen Asif:** Conceptualization, Writing - original draft, Writing - review & editing. **Abdul Ahad Sohail:** Writing - original draft, Writing - review & editing. **Saulat Hasnain Fatimi:** Conceptualization, Writing - review & editing.

## Declaration of competing interest

None.
